# Complete Genome Sequence of Pseudomonas syringae BIM B-268, a Plant Pathogen Strain Used for *In Vitro* Testing of Agricultural Bacteriophage Efficiency

**DOI:** 10.1128/MRA.00413-21

**Published:** 2021-07-01

**Authors:** Rustam M. Buzikov, Tatsiana A. Pilipchuk, Leonid N. Valentovich, Emilia I. Kalamiyets, Andrey M. Shadrin

**Affiliations:** a Laboratory of Bacteriophage Biology, G. K. Skryabin Institute of Biochemistry and Physiology of Microorganisms, Pushchino Scientific Center for Biological Research of the Russian Academy of Sciences, Federal Research Center, Pushchino, Moscow Oblast, Russia; b Institute of Microbiology of the National Academy of Sciences of Belarus, Minsk, Belarus; Loyola University Chicago

## Abstract

Pseudomonas syringae BIM B-268 is the strain used for *in vitro* testing of the efficiency of Multiphage, a bacteriophage-based biopesticide produced in Belarus. The genome sequence of this strain consists of a single circular chromosome harboring the genes encoding the ice nucleation protein, syringopeptin biosynthesis, and types III and VI secretion systems.

## ANNOUNCEMENT

Pseudomonas syringae strain BIM B-268 was isolated in the Minsk Region, Belarus, from a leaf from a blackcurrant bush (Ribes nigrum) and used for *in vitro* testing of the efficiency of the bacteriophage-based biopesticide Multiphage (1). This bacterial strain is a prospective model for studying bacteriophage-host interactions due to the synchronous adsorption of most of the phages of the Multiphage cocktail. The strain is deposited in the Belarussian Collection of Non-pathogenic Microorganisms of the Institute of Microbiology, National Academy of Sciences of Belarus (BIM), and kept under the following storage conditions: (i) freeze-dried with 10% fat-free powdered milk at 4°C and (ii) in 20% glycerol stock at −80°C. For DNA preparation, the strain was grown in 5 ml of GRM broth (Obolensk, Russia). The DNA was obtained using a bacterial DNA preparation kit (Jena Bioscience; catalog number PP-206S).

Oxford Nanopore (ONT) and Illumina sequencing were performed at BioSpark LCC. The KAPA HyperPlus kit (Roche) was used for MiSeq library preparation as recommended by the manufacturer. The SQK-LSK109 ligation kit and the EXP-NBD104 barcoding kit (ONT) were used for the Oxford Nanopore library preparation. The ONT library was prepared without DNA shearing or size selection. The library was sequenced on a MinION sequencer using a FLO-MIN106D R9.4.1 flow cell. The reads were called using Guppy v3.5.0 software in high accuracy mode. All listed software was used with default parameters.

A total of 2,054,402 Illumina MiSeq paired-end reads with a length of 251 nucleotides (nt) was obtained. For the assembly, 46,590 Oxford Nanopore reads with an average length of 2,137 nt (maximum read length, 122,273 nt) were used. The Nanopore reads were assembled into single contigs (coverage, 14×) using the Flye v2.8.3 assembler (2). Next, the Illumina reads (coverage, 120×) were used to correct Nanopore or assembly errors using Bowtie2 v2.4.2 (3) and Pilon v1.23 (4) software. The P. syringae BIM B-268 genome consists of a 6,018,586-bp circular chromosome (GC content, 59%). The average nucleotide identity between the type strain P. syringae KCTC 12500 and BIM B-268 was revealed using the ANIb tool from the JSpeciesWS website (5). The calculated 95.5% ANIb value (83.8% aligned) confirmed the taxonomic status of P. syringae BIM B-268.

The chromosome was annotated using the NCBI Prokaryotic Genome Annotation Pipeline v5.1 (6). In total, 5,165 genes were identified in the genome, including 5,004 protein-coding sequences (excluding pseudogenes) and five clusters of the 16S, 23S, and 5S rRNA genes, with one of them containing two copies of the 5S rRNA gene. Each rRNA gene cluster contained a tandem of isoleucine and alanine tRNA genes between the 16S and 23S rRNA genes. The whole genome of strain BIM B-268 includes 64 tRNA genes. A search for prophages using PHASTER (7) revealed two incomplete (2,758,936 to 2,788,722 and 4,960,194 to 4,976,915) and one intact (5,364,128 to 5,381,519) prophages with lengths of 29.7, 16.7, and 17.3 Kbp, respectively. The P. syringae BIM B-268 genome includes a number of plant pathogenicity factors: an ice nucleation protein (NCBI:protein accession number QQQ52141.1), two closely located genes of nonribosomal peptide synthetases (QQQ52950.1 and QQQ52948.1) (8), and clusters of the type III and type VI secretion system genes, which may give P. syringae a competitive advantage over other bacteria (9).

### Data availability.

This whole-genome project has been deposited in GenBank under accession number CP068034.2. The raw reads are available in the Sequence Read Archive under BioProject accession number PRJNA691121.
